# Niacin Alleviates Browning in Fresh-Cut Potatoes: Regulation of NADPH/NADH Levels Mediates ROS-Redox Homeostasis and the Ascorbate–Glutathione Cycle

**DOI:** 10.3390/foods15112020

**Published:** 2026-06-04

**Authors:** Jiaxuan Zheng, Mengyao Zhang, Ziyu Zhao, Ming Li, Ji Kang, Laifeng Lu, Liping Qiao, Xia Liu

**Affiliations:** State Key Laboratory of Food Nutrition and Safety, Key Laboratory of Food Nutrition and Safety, Ministry of Education, Tianjin Key Laboratory of Food Nutrition and Safety, College of Food Science and Engineering, Tianjin University of Science & Technology, Tianjin 300457, China; zhengjia_xuan@126.com (J.Z.); zmy061401@163.com (M.Z.); ziyuzhao1111@163.com (Z.Z.); liming_9809@163.com (M.L.); ji.kang@tust.edu.cn (J.K.); frank@tust.edu.cn (L.L.)

**Keywords:** enzymatic browning, niacin treatment, oxidant-redox regulation, pyridine nucleotide homeostasis, fresh-cut potato, preservation

## Abstract

Niacin contents vary significantly among fresh-cut potato cultivars with different browning sensitivities, whereas its role as a browning inhibitor for fresh-cut produce has not been previously reported. In this study, potato slices were soaked in distilled water (control) or 1% food-grade niacin solution for 5 min, then stored at 4 ± 1 °C for 8 days with sampling every 2 days for physiological and molecular analyses. In particular, the optimal niacin (1%) treatment showed higher brightness and lower color change than the control. The activities of polyphenol oxidase (PPO), peroxidase (POD), and phenylalanine ammonia lyase (PAL), and phenol content were reduced. Higher activities of superoxide dismutase (SOD) and catalase (CAT), and greater glutathione accumulation, were observed following niacin treatment. Meanwhile, lower levels of malondialdehyde and reactive oxygen species (ROS), and lower nicotinamide adenine dinucleotide phosphate oxidase (NOX) activity, indicated lower oxidant damage. The contents of NADP and NAD, and activities of nicotinamide adenine dinucleotide kinase (NADK) and glucose-6-phosphate dehydrogenase (G6PDH) were improved. Furthermore, the gene expression patterns of *StRBOH*, *StPPO*, and *StG6PDH* also supported the hypothesis that niacin regulates pyridine nucleotide and ROS homeostasis.

## 1. Introduction

Fresh-cut products have become popular worldwide in recent years due to their fresh, natural, nutritious, and convenient characteristics. Surface browning not only affects the shelf life of fresh-cut products but also reduces their market value [1]. Approximately 50% of food products that are sensitive to browning are wasted due to discoloration [2]. Hence, surface browning control is of great significance for fresh-cut products.

As is well known, surface browning of fresh-cut products is generally attributed to the conversion of phenolic substrates into quinones by polyphenol oxidase (PPO) or peroxidase (POD) under aerobic conditions, resulting in the accumulation of brown pigment [3,4]. Meanwhile, compared to fresh-cut fruit, the onset of browning in fresh-cut potatoes typically takes longer because phenolic substrates for browning accumulate in the substrate through phenylalanine ammonia lyase (PAL) catalysis during the first four days after injury [5]. More importantly, the accumulation of oxidant substances—such as malondialdehyde (MDA), superoxide anion ($O_{2}^{-}\cdot$), hydrogen peroxide (H_2_O_2_), and hydroxyl radical (·OH)—usually accompany browning development, which reflects the existence of excessive membrane oxidative stress after cutting. Hence, activation of the redox system, especially the regulation of reactive oxygen species (ROS) homeostasis, is vital for browning [6,7]. The enzymatic antioxidant system, including the activities of superoxide dismutase (SOD) and catalase (CAT), along with nonenzymatic antioxidants (such as glutathione, GSH), significantly contributes to alleviating oxidative damage [8,9]. Especially noteworthy, the sizes of the NAD(H) and NADP(H) pools are crucial for the redox state [10].

NADH is a key molecule that supplies reducing power to many catabolic pathways [11]. Research has shown that chlorine dioxide treatment alleviates pericarp browning by increasing nicotinamide adenine dinucleotide (NAD^+^) levels in longan fruit [12]. Activated nicotinamide adenine dinucleotide kinase (NADK) activity, upregulated levels of nicotinamide adenine dinucleotide phosphate (NADP^+^), and reduced nicotinamide adenine dinucleotide phosphate (NADPH) contribute to epibrassinolide’s ability to decrease chilling injury and the browning index in bananas [13]. Meanwhile, it was shown that upregulating glucose-6-phosphate dehydrogenase (G6PDH) helps postharvest fruit maintain higher antioxidant capacity and stress resistance by increasing intracellular NADPH levels [14]. Although niacin-mediated NAD metabolism and redox regulation have been well characterized, they remain unexplored in fresh-cut potato browning, mainly because prior studies focused on their nutritional functions rather than postharvest preservation applications. Therefore, the regulation of NAD(H) and NADP(H) can mediate browning of fresh-cut products during storage by balancing reactive oxygen species (ROS) and the redox biological system. As we know, niacin (NA) can be converted into NAD+ through the Preiss–Handler pathway in the body, and niacin and its derivative, nicotinamide, are forms of vitamin B3 and are known pyridine precursors found in a wide variety of foods [15]. NA benefits human health; in the context of niacin deficiency, “3D” diseases, namely, dermatitis, diarrhea, and dementia, can occur [15,16,17].

Our previous metabolomic analysis of cultivar differences showed that niacin accumulated to different levels (by a tenth of a percent) in two potato cultivars with different post-cut browning sensitivities [18]. However, whether niacin treatment can affect browning in the real-world setting is still unknown. Therefore, this study examined the influence of exogenous niacin treatment on surface browning in fresh-cut potatoes and investigated its effects on the browning reaction, antioxidant–oxidant system, and pyridine nucleotide homeostasis. We not only determined the activity levels of key enzymes and the contents of key substances but also investigated the expression of genes related to these processes.

## 2. Materials and Methods

### 2.1. Materials and Preparation

Fresh potatoes (*Solanum tuberosum*, cv Netherlands 15) in the mature stage and with uniform shape, medium size, and no physical damage were gathered from a local market in Tianjin, China, at full physiological maturity (determined by senesced haulms and >20% tuber dry-matter content). Standard conventional agricultural management was applied with routine low-dose chemical fertilizers and pesticides following local agricultural practices, and tubers were free of residual agrochemicals above safe thresholds. Tubers with uniform shape, medium size, no mechanical damage or disease symptoms were purchased from a local market within 7 days after harvest, then stored at 4 ± 1 °C. The natural enzymatic browning characteristics of untreated fresh-cut potato slices (timing and severity of browning development) were recorded as baseline reference throughout cold storage. Niacin, which was food grade and had a purity of 99%, was supplied by Jiangxi Brothers Pharmaceutical Co., Ltd., Jiangxi, China.

The potatoes were washed, peeled, and sliced into slices (5 mm). The slices were randomly assigned to two groups and soaked in solutions of distilled water (CK) and 1% niacin for 5 min. Afterward, potato samples were dried with gauze and packed in zip-lock bags (280 mm * 180 mm * 0.035 mm) and stored at 4 ± 1 °C for eight days. Then, the potato slices were randomly chosen from each group every two days for experimental index determinations.

### 2.2. Determination of Color and Appearance

To measure the color of the fresh-cut potato samples, a portable colorimeter (HP-200, Shanghai Gaozhi Precision Co., Ltd., Shanghai, China) was used. Determination indicators L*, a*, and b*, presented the values of brightness, red–green, and yellow–blue, respectively. The formula ΔE = $\sqrt{{∆L}^{2}+∆a^{2}+{∆b}^{2}}$ was applied to calculate the overall color change of fresh-cut potato slices [19]. Appearance changes were recorded by photographs.

### 2.3. Activities of PPO, POD, and PAL and Content of Total Phenolics

PPO and POD enzymatic activities were measured by a previously reported method [20]. The crude extracts used in the PPO and POD enzyme activity assays were prepared by homogenizing the fresh-cut potatoes (2 g) with 5.0 mL extraction buffer (containing 1 mmol L^−1^ PEG, 4% PVPP, and 1% Triton X-100) and then centrifuging at 12,000× *g* for 30 min.

To test the POD activity, 0.5 mol L^−1^ H_2_O_2_ (200 μL) was added to a mixture containing guaiacol solution (25 mmol L^−1^, 3.0 mL) and the above crude extract solution (0.5 mL) for a rapid reaction. The absorbance values were detected at 420 nm every 60 s, and POD activity was indicated as 10^3^ U kg^−1^, where $U={∆OD}_{420}/min$, the increase in absorbance value per minute at 420 nm.

To detect PPO activity, acetic acid-sodium acetate buffer (0.05 mol/L, 4.0 mL), 0.05 mol/L catechol solution (1.0 mL), and enzyme extract (100 μL) were quickly mixed thoroughly. Afterward, the absorbance was recorded. PPO activity was given in 10^3^ U kg^−1^, where $U={∆OD}_{470}/min$.

The method for measuring PAL activity was as described by a previously reported method [21]. The tissue of potato samples (2 g) was mixed evenly with the extraction buffer (5.0 mL) (containing 4% PVP, 2 mmol/L EDTA, and 5 mmol/L β-mercaptoethanol) and then centrifuged (12,000× *g*, 30 min). To evaluate PAL activity, a mixture consisting of 3.0 mL borate buffer (50 mmol/L, pH 8.8) and 0.5 mL L-phenylalanine (50 mmol L^−1^) was reacted (37 °C, 10 min). Then, 0.5 mL enzyme extract was added. After the initial absorbance value at 290 nm was quickly tested, the above mixtures were kept at 37 °C for 1 h and then tested again to record the final absorbance. The PAL activity was defined as 10^3^ U kg^−1^ of fresh weight, and one unit (U) was an increase of 0.01 in the absorbance value per hour at 290 nm.

The total phenolic content was assayed by using published methods with modifications [22]. Crude enzyme extracts were obtained from the fresh-cut sample (2 g) after homogenization with 5 mL ethanol and centrifugation (12,000× *g*, 10 min). A reaction system, which contained 0.25 mL supernatant enzyme solution, 2.0 mL distilled water, 0.8 mL Na_2_CO_3_ (20%, *w*/*w*), and 0.25 mL Folin-phenol Reagent, was reacted in the dark for 25 min to obtain the absorbance at 760 nm. The total phenolic content was calculated as g kg^−1^ of fresh weight.

### 2.4. Content of MDA and H_2_O_2_, Rate of $O_{2}^{-}\cdot$ Production, and NOX Activity

The method used to detect the MDA content was based on Hassan, Ali [23]. Fresh samples (1 g) were homogenized with 10% trichloroacetic acid (5 mL, *w*/*w*) and centrifuged for 20 min (12,000× *g*). Mixtures containing 2 mL extract of samples and 2 mL 0.67% thiobarbituric acid (*w*/*w*) were thoroughly boiled for 20 min. Soon after centrifuging the above cooled mixtures (12,000× *g*, 20 min), the absorbance was tested at 450, 532, and 600 nm. Finally, the content of fresh weight was represented as μmol kg^−1^.

In line with previous research, the H_2_O_2_ content was detected [24]. To obtain the enzyme extract, the mixed solution, which consisted of the fresh sample (2 g) and precooled acetone (5 mL), was centrifuged (12,000× *g*, 20 min). The mixture of 1.0 mL enzyme extract, 0.1 mL titanium tetrachloride-hydrochloric acid, and 0.2 mL concentrated ammonia was centrifuged (12,000× *g*, 20 min). Precooled acetone was used to wash the precipitate several times to remove the pigment. The precipitate was dissolved by sulfuric acid. Finally, the absorbance of the resulting solution at 412 nm was detected, and the H_2_O_2_ content of the fresh weight was given in mol kg^−1^.

The $O_{2}^{-}\cdot$ production rate was estimated as a published report with some modifications [25]. A well-mixed solution containing 1.0 mL enzyme extract, sodium phosphate buffer (1.0 mL, 50 mmol/L, pH 7.8), and hydroxylamine hydrochloride (1.0 mL, 1 mmol/L) was incubated (25 °C,1 h). Then, the reaction system of the above incubated mixture, 1.0 mL p-aminobenzenesulfonic acid (17 mmol/L) and 1.0 mL α-naphthylamine (7 mmol/L), was incubated again for 25 min (25 °C) to obtain the absorbance at 530 nm. The rate of $O_{2}^{-}\cdot$ production was defined in μmol min^−1^ kg^−1^.

NADPH oxidase (NOX) activity was tested by using a reported method [26] with slight modifications. The fresh sample (1 g) was mixed with MES-Tris buffer (5 mL, 25 mmol/L, pH 7.8; containing 3 mmol/L EDTA, 0.25 mol/L sucrose, 5 mmol/L DTT, 1 mmol/L PMSF, and 1% PVPP) and centrifuged (12,000× *g*, 25 min). The precipitate was dissolved in 2.5 mL of 5 mmol/L MES-Tris buffer (pH 7.8, containing 0.25 mol/L sucrose, 5 mmol/L KCl, 1 mmol/L PMSF, and 5 mmol/L DTT) for later use. Then, absorbance was recorded at 470 nm for the reaction system, which contained 2 mL of 50 mmol/L Tris-HCl buffer (pH 7.5), 0.2 mL of 0.5 mmol/L XTT solution, 0.2 mL of the above enzyme extract, and 0.1 mL of NADPH (100 μmol/L). Finally, the NOX activity was represented as 10^3^ U kg^−1^, and one unit (U) was regarded as 0.01 ∆A_470_ min^−1^.

### 2.5. SOD and CAT Activities, GSH Content, and Antioxidant Capacity

The inhibitory capacity of nitrogen blue tetrazolium (NBT) photoreduction was applied to evaluate SOD activity [27]. The reaction system, composed of sodium phosphate buffer (1.7 mL, 50 mmol/L, pH 7.8), methionine solution (0.3 mL, 0.13 mmol/L), nitrogen blue tetrazolium solution (0.3 mL, 0.75 mmol/L), EDTA-Na_2_ solution (0.3 mL, 0.1 mmol/L), supernatant (0.1 mL), and riboflavin solution (0.3 mL, 0.1 mmol/L), was placed under fluorescent light to react for 15 min. Moreover, two test tubes containing the above reaction solution (0.1 mL distilled water instead of the supernatant) were placed under fluorescent light and the dark for 15 min. Absorbance at 530 nm was assayed, and SOD activity was defined in 10^3^ U kg^−1^ of fresh weight. One unit (U) was defined as 50% inhibition of NBT photoreduction min^−1^.

CAT activity was estimated by a published method [28]. Fresh samples of 2 g and 5 mL of sodium phosphate buffer (0.1 mol/L, pH 7.5; involving 5 mmol/L DTT, 5% PVP) were centrifuged (12,000× *g*, 30 min). The reaction was rapidly initiated when 2.9 mL of H_2_O_2_ solution and 100 μL of supernatant were mixed. Finally, absorbance was recorded, and CAT activity was represented as the fresh weight of 10^3^ U kg^−1^. One decreased unit (U) was regarded as 0.01 ∆A_240_ min^−1^.

The GSH content was detected according to a published method [29]. Fresh tissue samples (2 g) were mixed with precooled trichloroacetic acid (5 mL, 50 g/L) at 4 °C, then centrifuged at 12,000× g for 30 min to obtain enzyme extracts. After 1.0 mL of enzyme extract and 0.1 mol/L sodium phosphate buffer (1.0 mL, pH 7.7) were added to two test tubes, 0.5 mL of dithionitrobenzoic acid was added to one tube, and sodium phosphate buffer (0.5 mL, 0.1 mol/L, pH 6.8) was added to the other tube. Finally, the abovementioned reaction tubes were incubated (25 °C, 10 min) to obtain the absorbance at 412 nm. The GSH content was defined as mmol kg^−1^.

The antioxidant capacity of the samples was calculated according to the scavenging ability of 2,2′-diphenyl-1-picrylhydrazyl (DPPH) free radicals via a published method [30]. The A_r_, A_s_, and A_0_ absorbance values were obtained at 517 nm. Finally, DPPH (%) was calculated as $\left[1-(A_{S}-A_{r})/A_{0}\right]\times 100$.

### 2.6. Content of Pyridine Nucleotides and Activity of NADK and G6PDH

The content of pyridine nucleotides was measured by applying a published method [31]. Fresh samples (1 g) were homogenized with NaOH solution (5 mL, 0.1 mol/L) for NAD(P)H determination or HCl solution (5 mL, 0.1 mol/L) for NAD(P)^+^ determination. The homogenates were centrifuged (10,000× *g*, 10 min) after a boiling water bath (5 min). The supernatants were neutralized with NaOH or HCl solution for the next procedure.

A mixture of 50 μL supernatant, 450 μL 0.1 mol/L NaCl, and 300 μL Tricine-NaOH (0.1 mol/L, containing 40 mmol L^−1^ EDTA, 16.6 mmol/L^1^ PES, 4.2 mmol/L MTT, 25 mmol L^−1^ 6-phosphoglucose or 5 mol L^−1^ ethanol) was incubated (37 °C, 5 min) for NADP(H) or NAD(H) determination. Then, 50 μL of ethanol dehydrogenase solution (for NAD^+^ and NADH assays) or 6-phosphoglucose dehydrogenase solution (for NADP^+^ and NADPH assays) was added. After incubation (37 °C, 45 min), the reaction was terminated with NaCl solution (600 μL, 6 mol/L), and the mixture was centrifuged (10,000× *g*, 10 min) to collect the precipitate, which was then dissolved in ethanol (4 mL, 96%, *v*/*w*). Absorbance was measured at 570 nm.

NADK activity was tested with the published method [13]. Fresh potato samples (1 g) were homogenized with Tris-HCl buffer (5 mL, pH 7.8, 50 mmol/L) and centrifuged at 10,000× *g* (30 min). Then, a mixture of 0.1 mL supernatant and 0.4 mL of 0.1 mol/L Tris-HCl buffer (pH 7.8, containing 10 mmol/L MgCl_2_, 2 mmol/L NAD^+^, 3 mmol/L ATP) was incubated at 37 °C (1 h). After incubation, the above mixtures were boiled for 5 min to terminate the reaction and then centrifuged (8000× *g*, 10 min). Finally, the activity of NADK was expressed as μmol min^−1^ kg^−1^ NADP^+^.

The G6PDH Assay Kit (Spectrophotometer, Lot. No. 20220629, Wuhan, China) was applied to test G6PDH activity by following the manufacturer’s instructions. The activity of G6PDH on a fresh weight basis was defined as 10^3^ U kg^−1^, and one unit (U) was regarded as 0.1 ∆A_3400_ min^−1^.

### 2.7. Analysis of qRT-PCR

Total RNA was extracted with RNAiso Plus (Code No. 9108, Takara Bio, Beijing, China) based on the product’s instructions. cDNA was synthesized with HiScript^®^ III RT SuperMix for qPCR (Vazyme Biotech Co., Ltd., Tianjin, China), and qRT-PCR was conducted with 2× ChamQ Universal SYBR qPCR Master Mix (Vazyme Biotech Co., Ltd., Tianjin, China) following the manufacturer’s instructions. The specific primers listed in Table 1 were designed online with Primer3 Plus. Quantitative real-time polymerase chain reaction (qRT-PCR) was conducted by employing a MyCycler^TM^ Thermal Cycler (Bio-Rad, Herculaneum, CA, USA) under the following thermal cycling conditions: 95 °C for 30 s for the initial denaturation; 95 °C for 5 s with 40 cycles, 60 °C for 15 s for annealing, and 60 °C for 1 min; and 95 °C for 15 s.

### 2.8. Statistical Analysis

A completely randomized experiment was designed. All treatments were replicated at least three times to obtain data, which were analyzed using SPSS 26. Normality and homogeneity of variance were checked prior to analysis to validate parametric assumptions. Data are expressed as means ± standard deviations. The results were analyzed by *t*-test and calculated at the 95% confidence level (*p* < 0.05).

## 3. Results and Discussion

### 3.1. The Appearance and Color Change

**Figure 1:**
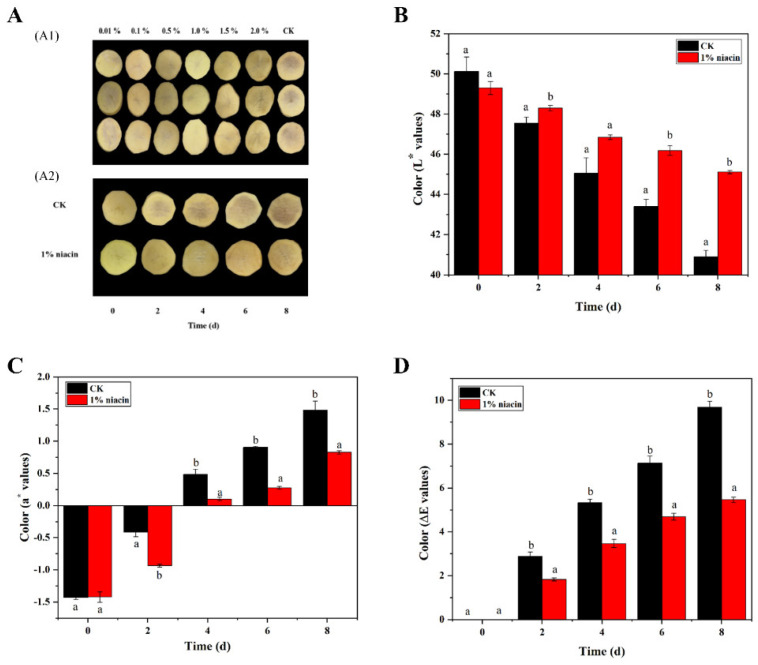
The appearance and color of fresh-cut potatoes under CK and niacin treatment (CK: distilled water; 0.01%, 0.01%, 0.1%, 0.5%, 1.0%, 1.5%, 2.0% niacin immersion treatment) in the preliminary study and further experiments during storage at 4 °C for 8 days. (**A**) Photographs ((**A1**), the appearance of samples in the preliminary study on day 2; (**A2**), the appearance of samples under CK and 1% niacin treatment during the storage of 8 days), (**B**) L* values, (**C**) a* values, and (**D**) ΔE values. Data are presented as means ± standard deviations from three replications, and different lowercase letters indicate significant differences among treatments at *p* < 0.05.

Color is part of the first impression of food and plays a key role in affecting consumer decisions. A preliminary study was conducted to determine the optimal niacin concentration in two steps. Firstly, the concentration range of 0.01%, 0.1%, and 1.0% were determined, and then 0.5%, 1%, 1.5%, and 2.0% were verified. The appearance of the niacin-treated slices was better than that of the control slices on day 2 (Figure 1(A1)), and the 1.0% niacin treatment produced the best appearance. However, the sample with a concentration of 2.0% was deteriorated. The 1.0% niacin-treated slices showed a lighter color, and an approximately 10.3% higher L* value was observed after 8 days of storage (Figure 1(A2,B)). Meanwhile, the a* values differed by 30% between the two groups at the end of storage (Figure 1C). Notably, the overall color difference ΔE value of the niacin-treated sample was only 50% of that of the control after 8 days of storage (Figure 1D). Overall, the niacin treatment helped the slices maintain a better color quality.

Enzymatic browning is a troublesome issue for fresh-cut potatoes [32]. Here, the results showed that potato slices treated with niacin maintained lower a* values and higher L* values, while minimizing color changes (ΔE). It is believable that niacin has an anti-browning effect, helping maintain better appearance quality and delaying the browning of fresh-cut potatoes. The core of enzymatic browning is phenolic oxidation caused by ROS accumulation. Niacin, a form of vitamin B3, could activate antioxidant enzymes and increase the expression levels of stress resistance genes through NAD+ metabolism [33,34].

### 3.2. Key Factors in the Browning Reaction: PPO, POD, and PAL Activities and Total Phenolic Content

**Figure 2:**
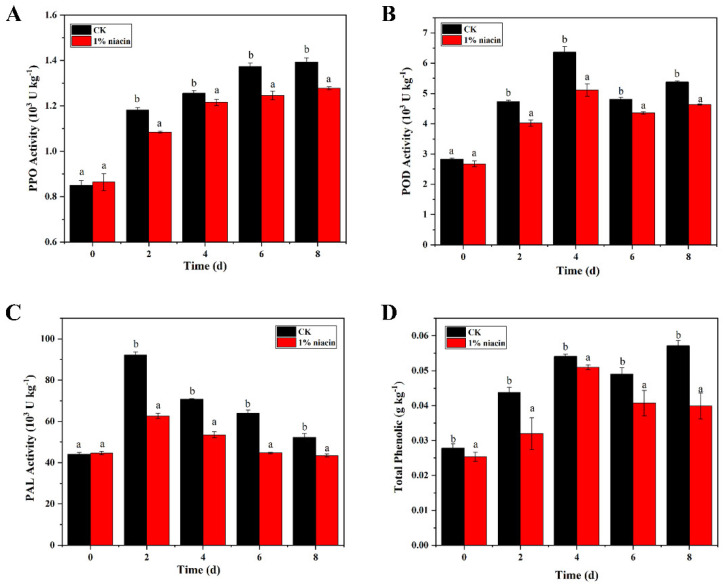
Changes in key enzyme activities in the browning reaction and phenol metabolism of potato slices during storage at 4 °C for 8 days after 1% niacin immersion and CK treatment. (**A**) PPO activity, (**B**) POD activity, (**C**) PAL activity, and (**D**) total phenolic content. Data are presented as means ± standard deviations from three replications, and different lowercase letters indicate significant differences among treatments at *p* < 0.05.

Cell damage after cutting leads to contact between PPO and the substrate, catalyzing phenolic oxidation. As widely reported, the PPO-POD-PAL cascade constitutes the core regulatory pathway governing enzymatic browning in fresh-cut potatoes, in which PAL mediates phenolic biosynthesis, and PPO/POD jointly catalyze phenolic oxidation. PPO activity increased with preservation time, and niacin treatment activity was approximately 10.2% lower than that of CK on day 8 (Figure 2A). Niacin treatment appeared to maintain this steady activity from day 4, while the PPO activity in the untreated group tended to stabilize from day 6. Collaborating with PPO, POD is also known to aggravate browning by participating in the oxidation of phenolic substances, which peaked on day 4. The POD activities of the niacin-treated slices were lower than those of the control treatment at all time points (Figure 2B). PAL activity, another key enzyme involved in browning development, showed an initial increase followed by a decrease during storage, peaking on day 2, at which point it was approximately 47.1% lower in niacin-treated slices than in controls. (Figure 2C). The enzymatic browning substrate, phenolic content, was 30.3% lower in the niacin-treated slices than in the CK slices at the end of storage (Figure 2D).

Enzymatic browning induced by the PPO-POD-PAL cascade is a common issue that has been widely studied in fresh-cut mushrooms, fruits, and vegetables for decades. Our study further revealed that niacin-mediated regulation of pyridine nucleotides confers unique anti-browning effects in fresh-cut potatoes. The PAL, PPO, POD activity, and phenolic content are key factors in the browning reaction [35]. Here, niacin treatment could suppress the activities of PPO, POD, and PAL, as well as the phenolic content in potato slices. All of these factors led to lower potato surface browning under niacin treatment, which was inconsistent with the anti-browning models [36]. The cause might be indirect, modulating the cell’s physiological and biochemical status, because high levels of NADPH help maintain the Redox State of GSH and abscisic acid within cells [36,37,38,39].

### 3.3. ROS-Redox Homeostasis

**Figure 3:**
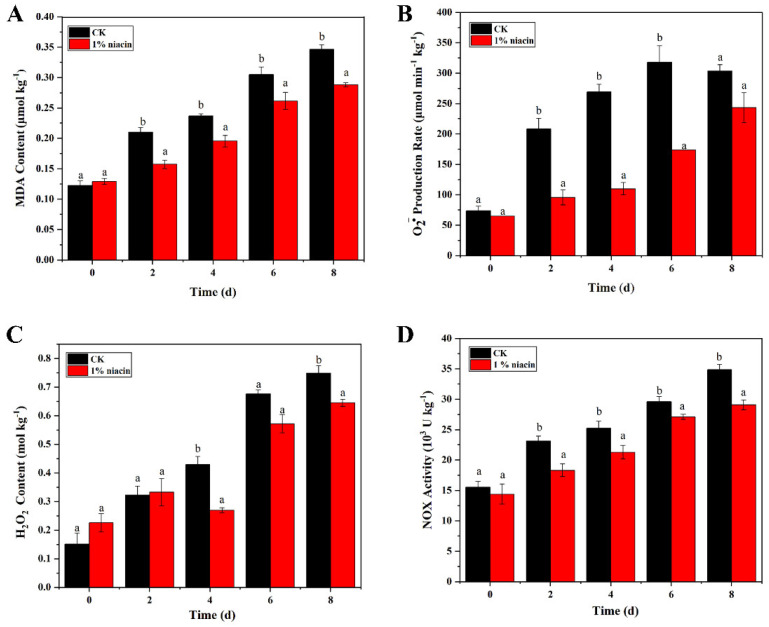
Oxidative damage factors in potatoes during storage at 4 °C for 8 days after 1% niacin immersion and CK treatment. (**A**) MDA content, (**B**) $O_{2}^{-}\cdot$ production rate, (**C**) H_2_O_2_ content, and (**D**) NOX activity. Data are presented as means ± standard deviations from three replications, and different lowercase letters indicate significant differences among treatments at *p* < 0.05.

Typically, serious oxidant damage is concurrent with fresh-cut processing and enzymatic browning development. However, moderate levels of ROS act as signaling molecules and participate in physiological processes, from cell proliferation to differentiation [40]. MDA, a significant indicator of membrane damage produced by lipid peroxidation, increased with prolonged storage (Figure 3A). While niacin-treated slices showed approximately 16.8% less than in the CK on day 8. Meanwhile, the accumulation of ROS—consisting of H_2_O_2_, $O_{2}^{-}\cdot$, and OH·—generally reflects the occurrence of oxidative damage, showed not only the delay in the peak time of $O_{2}^{-}\cdot$ accumulation, but also reduced the $O_{2}^{-}\cdot$ content in the niacin treatment (Figure 3B). Even more importantly, nearly 45.5% less $O_{2}^{-}\cdot$ was produced on day 6 in the niacin-treated samples, which was even less than that of the control samples on day 2. A similar result was found for H_2_O_2_ content, with niacin leading to less accumulation (Figure 3C). NOX, which is responsible for generating reactive oxygen species within cells, the cutting process of fresh fruits and vegetables enhanced NOX activity and induced a higher ROS level, further led to more MDA accumulation, and lower antioxidant levels. The NOX activity increased with storage, and approximately one-sixth less NOX activity was observed on day 8 after niacin treatment than that in the CK group (Figure 3D).

**Figure 4:**
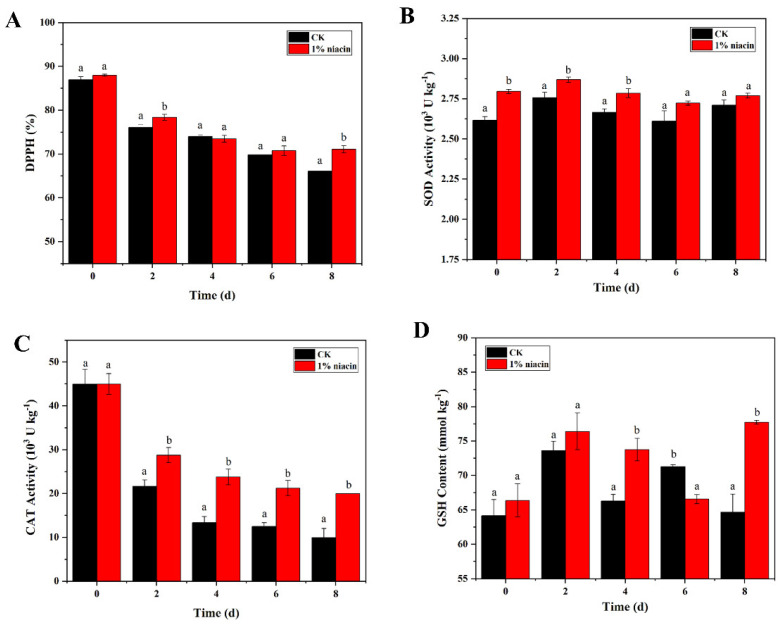
The antioxidant level of potato slices during storage at 4 °C for 8 days after 1% niacin immersion and CK treatment. (**A**) DPPH scavenging capacity, (**B**) SOD activity, (**C**) CAT activity, and (**D**) GSH content. Data are presented as means ± standard deviations from three replications, and different lowercase letters indicate significant differences among treatments at *p* < 0.05.

The total antioxidant capacity, termed the free radical DPPH scavenging capacity, was 7.6% higher in samples of niacin treatment than CK slices on day 8 (Figure 4A). Antioxidant enzyme activity of SOD was enhanced in niacin treatment during the first four days (Figure 4B). During storage, CAT activity decreased dramatically, then remained steady, and a higher level was found in the niacin-treated slices (Figure 4C). GSH, an important antioxidant involved in ROS homeostasis and crucial for browning regulation, fluctuated during storage, and more GSH was produced after niacin treatment (Figure 4D).

Overall, ROS-redox homeostasis was better maintained after niacin treatment. As in existing studies, less browning occurs; for example, exogenous sodium isoascorbate treatment delayed the browning of fresh-cut mushrooms and prolonged their shelf life by increasing the activity of antioxidant enzymes (SOD, CAT, etc.), reducing the levels of MDA and ROS [41,42]. Salicylic acid could also delay the browning of fresh-cut pomegranate, mainly due to improvements in DPPH radical scavenging and enzyme activities, such as CAT, SOD, APX, and GR, along with lower H_2_O_2_ accumulation [43]. Hence, the application of niacin could enhance ROS-scavenging capacity and reduce lipid peroxidation product formation, thereby alleviating oxidative damage and browning.

### 3.4. Pyridine Nucleotide Metabolism

**Figure 5:**
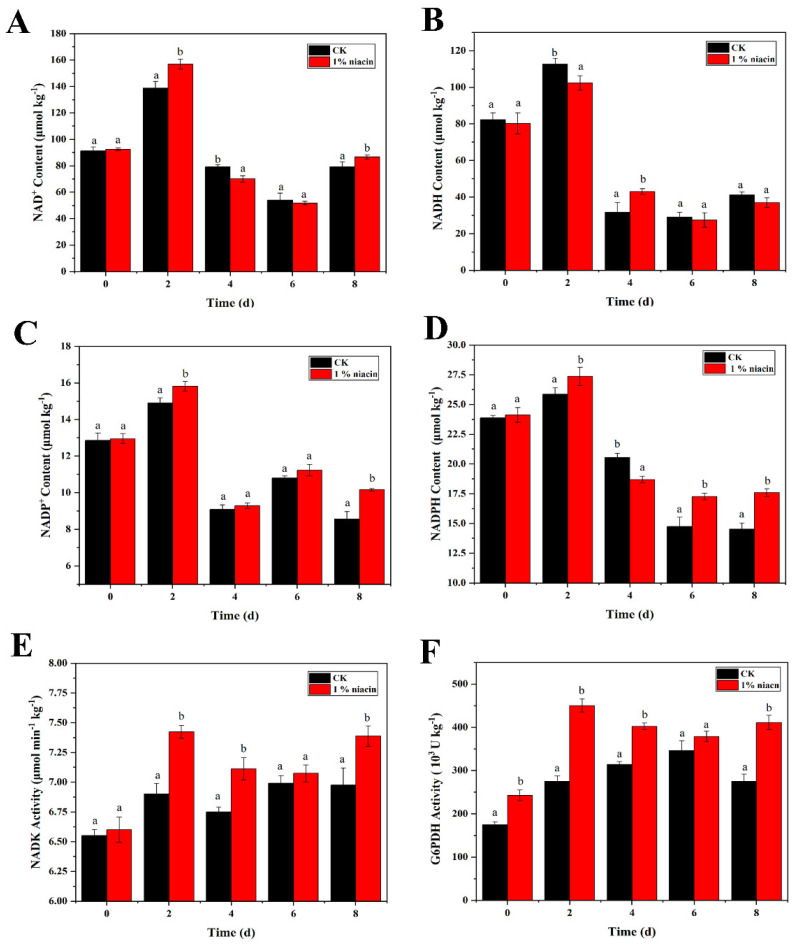
The pyridine nucleotide level of potato slices during storage at 4 °C for 8 days after 1% niacin immersion and CK treatment. (**A**) NAD^+^ content, (**B**) NADH content, (**C**) NADP^+^ content, (**D**) NADPH content, (**E**) NADK activity, and (**F**) G6PDH activity. Data are presented as means ± standard deviations from three replications, and different lowercase letters indicate significant differences among treatments at *p* < 0.05.

The fluctuations in NAD+ and NADP+ not only reflect the metabolic network activity and redox states of plant cells but also control cell redox status and signaling functions and significantly impact stress tolerance [44]. The levels of NAD+ and NADH peaked on day 2, with approximately 13.1% and 9.5% more NAD+ produced on days 2 and 8 after niacin application, respectively (Figure 5A,B). The NADP^+^ content fluctuated during preservation, and the niacin treatment produced 6.1% and 15.7% increases on days 2 and 8, respectively (Figure 5C). The NADPH level peaked on day 2 and then dropped. At the end of storage, approximately one-fifth more NADPH was produced on day 6 and day 8 in the niacin-treated slices (Figure 5D). Moreover, NADK and G6PDH are crucial enzymes in pyridine nucleotide metabolism and are responsible for phosphorylating and converting NADP^+^ to NADPH [13,45]. Figure 5E,F show that niacin application enhanced the activity of NADK and G6PDH. The G6PDH activity of the niacin treatment sample was approximately 1.6 and 1.5 times that of the control on days 2 and 8, respectively.

ROS is largely dependent on NAD(P)H in plant cells [45]. NAD^+^ and NADP^+^, as ubiquitous electron carriers, modulate ROS-redox homeostasis via electron transfer during reduction–oxidation [46]. NADPH is typically a significant cellular redox substance that helps reactivate the CAT enzyme quenched by H_2_O_2_ and provides reducing power to ensure the regeneration of GSH [45]. Here, niacin-induced elevation in NADPH content was significantly positively correlated with increased CAT activity, as NADPH supplies reducing equivalents to support CAT-mediated ROS scavenging. Sprayed niacin treatment boosted NADPH and NADP^+^ levels and reduced ROS ($O_{2}^{-}\cdot$ and H_2_O_2_) accumulation in kiwifruit leaves and may enhance stress tolerance during the short term [47]. In fact, many studies have shown a close correlation between pyridine nucleotide levels and the processes of plant organs aging [10]. It can also be simply understood that the higher NADP/NAD^+^ level, the higher stress resistance. From this perspective, it is reasonable to believe that the activation of NADK and G6PDH and the upregulation of NAD^+^, NADP^+^, and NADPH after niacin application may contribute to pyridine nucleotide homeostasis, synergistically help modulate the ROS-redox balance, and thus alleviate surface browning. Notably, the anti-browning effect of niacin differs from that of conventional general antioxidants. While common antioxidants mainly scavenge ROS and inhibit browning-related enzymes non-specifically, niacin exerts its unique function by regulating NAD-dependent pyridine nucleotide metabolism, activating NADK and G6PDH, and maintaining intracellular NADPH-GSH redox homeostasis, which represents a niacin-specific regulatory pathway rather than a general antioxidant response [48].

### 3.5. Relative Gene Expression Levels of Oxidative Burst-Related Factors, Browning Reaction, and Reducing Power Generation

**Figure 6:**
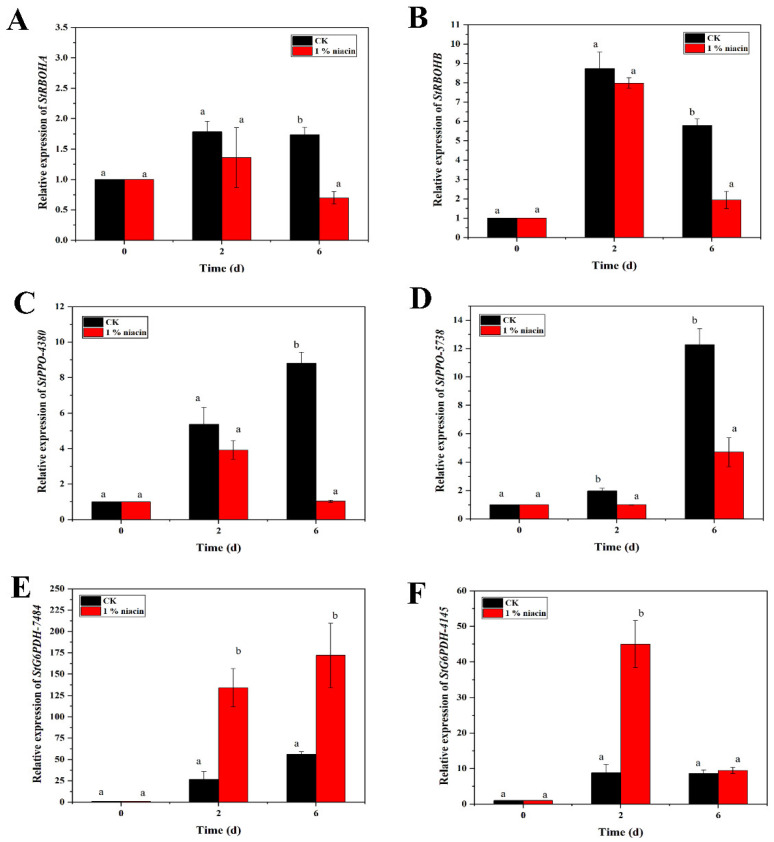
Relative gene expression levels of oxidative burst factors and the browning reaction, and the reducing power generation of potato slices on day 2, day 6, and day 8 of storage at 4 °C after 1% niacin immersion and CK treatment. (**A**) Relative expression of StRBOHA, (**B**) Relative expression of StRBOHB, (**C**) Relative expression of *StPPO-4380*, (**D**) Relative expression of *StPPO-5738*, (**E**) Relative expression of StG6PDH-7484, and (**F**) Relative expression of *StG6PDH-4145*. Data are presented as means ± standard deviations from three replications, and different lowercase letters indicate significant differences among treatments at *p* < 0.05.

The *RBOH* family encodes NADPH oxidase (NOX), which catalyzes the conversion of O_2_ to $O_{2}^{-}\cdot$ and regulates ROS levels [49]. As illustrated in Figure 6A,B, niacin treatment downregulated *StRBOHA* and *StRBOHB* expression levels on day 6, which were 59.9% and 66.5% lower than those in the control slices, respectively. Niacin treatment controlled the expression of *StPPO-4380* and *StPPO-5738* (Figure 6C,D). The expression of *StPPO-4380* and *StPPO-5738* in the control group was approximately 7.6 and 1.6 times higher than that of slices with niacin treatment on day 6, respectively. Importantly, niacin application enhanced *StG6PDH-7484* expression by 4.0- and 2.1-fold on day 2 and day 6, and 4.1-fold expression of *StG6PDH-4145* was found compared to the control on day 2 (Figure 6E,F).

Determining relevant gene expression levels and key enzyme activities may further elucidate the mechanism of action. A previous study proved that diphenyleneiodonium treatment suppressed phenylpropanoid metabolism in potato tubers, reduced the generation of $O_{2}^{-}\cdot$ and H_2_O_2_, and inhibited *StRBOH* expression and NOX activity [49]. Consistently, the downregulation of *StRBOH* gene expression was in line with the lower NOX activity in the niacin-treated slices (Figure 3D and Figure 6A,B). Another study reported that CO_2_ treatment not only delayed browning development but also increased G6PDH and 6PGDH activity and gene expression in fresh-cut pear [45]. A similar result was also obtained for G6PDH enzyme activity and gene expression in this study (Figure 5F and Figure 6E,F). Our previous transcriptomic analysis of cultivar differences showed that *StPPO-4380* and *StPPO-5738* were differentially expressed between two cultivars with different post-cut browning sensitivities [18]. Therefore, the consistent results for gene expression and key enzyme activities further proved that niacin treatment regulated browning development by promoting pyridine nucleotide metabolism and ROS-redox homeostasis.

## 4. Conclusions

Here, the anti-browning effect of exogenous niacin was first confirmed. The optimal concentration was 1%, which resulted in brighter potato slices throughout cold storage. Lower PPO, POD, and PAL activities and fewer phenolic substances were found. Meanwhile, the redox system was enhanced, accompanied by higher SOD, CAT, DPPH-scavenging capacity, and GSH levels. Moreover, oxidative damage was significantly alleviated owing to lower contents of H_2_O_2_, $O_{2}^{-}\cdot$, and MDA, along with lower NOX activity. Finally, pyridine nucleotide homeostasis was modulated due to the enhancement of NADK, G6PDH, NAD^+^, NADP^+^, and NADPH levels in niacin-treated slices. The downregulation of *StRBOHA*, *StRBOHB*, *StPPO-4380*, and *StPPO-5738*, along with the upregulation of *StG6PDH-7484* and *StG6PDH-4145*, verified the hypothesis that niacin application regulates pyridine nucleotide and ROS homeostasis. This is the first report of the benefits of niacin for anti-browning, by regulating pyridine nucleotide homeostasis and ROS-redox balance. As an essential human nutrient with generally recognized safety as a food additive, niacin treatment at the tested concentration shows promising potential for industrial-scale application of fresh-cut potato preservation. However, further sensory and commercial processing evaluations are still needed. Notably, this study has certain limitations: it was conducted using a single potato cultivar under laboratory cold storage conditions, and the detailed molecular regulatory network of niacin-mediated anti-browning remains to be further explored.

## Figures and Tables

**Table 1:** Primer sequences for real-time quantitative PCR (qRT-PCR) analysis.

Gene	Forward Primer (5′-3′)	Reverse Primer (5′-3′)
*StRBOHA* (LOC102579392)	GTTTACCTGGGCATGAACGC	CTCCACCAATACCGACTCCG
*StRBOHB* (LOC102603596)	TCTTCATTTCAGTGGCATCCGT	GTTGGAGGCTCACAGACCTT
*StPPO-4380* (LOC102604380)	TGGCTTTTCTTCCCGTTCCA	TTCACCACGCCTTTCGTCAT
*StPPO-5738* (LOC102605738)	CTTCTTCAACCACCACCACTT	GGAGACCACCAACAAGAGTAGA
*StG6PDH-7484* (LOC102597484)	AAGATGGTGCTGTTGTGGCT	CAGTCATCTTACTCCGGGCA
*StG6PDH-4145* (LOC102594145)	TCTGCGCCTACAACCTGATG	GGTCACTCCGGTCAAGTCTC
ef1α	GCTGCTGTAACAAGATGGATG	CAGGGTTGTAACCGACCTTCT

## Data Availability

The original contributions presented in the study are included in the article; further inquiries can be directed to the corresponding authors.
